# Incidence and physiological mechanism of carboplatin-induced electrolyte abnormality among patients with non-small cell lung cancer

**DOI:** 10.18632/oncotarget.12813

**Published:** 2016-10-21

**Authors:** Yushui Ma, Likun Hou, Fei Yu, Gaixia Lu, Shanshan Qin, Ruting Xie, Huiqiong Yang, Tingmiao Wu, Pei Luo, Li Chai, Zhongwei Lv, Xiaodong Peng, Chunyan Wu, Da Fu

**Affiliations:** ^1^ Department of Nuclear Medicine, Shanghai Tenth Peoples Hospital, Tongji University School of Medicine, Shanghai, China; ^2^ Shanghai Engineering Research Center of Molecular Therapeutics and New Drug Development, College of Chemistry and Molecular Engineering, East China Normal University, Shanghai, China; ^3^ Department of Pathology, Shanghai Pulmonary Hospital, Tongji University School of Medicine, Shanghai, China; ^4^ Department of Pathology, Shanghai Tenth Peoples Hospital, Tongji University School of Medicine, Shanghai, China; ^5^ Department of Oncology, the First Affiliated Hospital of Nanchang University. Nanchang, China; ^6^ Central Laboratory for Medical Research, Shanghai Tenth Peoples Hospital, Tongji University School of Medicine, Shanghai, China

**Keywords:** carboplatin, hyponatremia, hypokalemia, dehydration, FDA adverse event reporting system

## Abstract

To clarify the association between carboplatin and electrolyte abnormality, a pooled-analysis was performed with the adverse event reports of non-small cell lung cancer patients. A total of 19901 adverse events were retrieved from the FDA Adverse Event Reporting System (FAERS). Pooled reporting odds ratios (RORs) and 95% CIs suggested that carboplatin was significantly associated with hyponatremia (pooled ROR = 1.57, 95% CI 1.182.09, *P* = 1.99 × 10^-3^) and hypokalemia (pooled ROR = 2.37, 95% CI 1.803.10, *P* = 5.24 × 10^-10^) as compared to other therapies. In addition, we found that dehydration was frequently concurrent with carboplatin therapy (pooled ROR = 2.01, 95% CI 1.522.66, *P* = 8.37 × 10^-7^), which may prompt excessive water ingestion and decrease serum electrolyte concentrations. This information has not been mentioned in the FDA-approved drug label and could help explain the physiological mechanism of carboplatin-induced electrolyte abnormality. In conclusion, the above results will facilitate clinical management and prompt intervention of life-threatening electrolyte imbalance in the course of cancer treatment.

## INTRODUCTION

Drug-induced electrolyte abnormality has been widely observed in hospitalized patients [1, 2]. Hyponatremia (defined as serum sodium concentration lower than 135 mmol/L) and hypokalemia (defined as serum potassium concentration lower than 3.5 mmol/L) are two of the most common forms of electrolyte abnormality. In clinical practice, electrolyte abnormality is found to cause various symptoms and to be associated with negative consequences regarding morbidity and mortality [3, 4]. In the course of treatment with specific drugs, electrolyte abnormality may develop either symptomatically or asymptomatically [5]. Since it is difficult for clinicians to pay constant attention to drug reactions, drug-induced electrolyte abnormality may be underdiagnosed in some cases. Therefore, thorough awareness of the adverse effects of certain drugs on serum sodium and potassium levels is of great importance for effective prevention of potentially life-threatening electrolyte disturbance.

Carboplatin is a chemotherapy drug broadly used against a variety of cancers, such as non-small cell lung cancer [6]. As a platinum-based antineoplastic agent interfering with DNA repair, carboplatin gained increasing acceptance in clinical treatment due to greatly reduced toxicity profile compared to its parent compound cisplatin [7, 8]. However, in recent years, isolated events of hyponatremia and hypokalemia following carboplatin administration have been reported [9, 10]. Therefore, analysis based on larger samples is necessary to confirm the potential risks of carboplatin. However, obtaining and interpreting large-scale data has always been a challenge in clinical studies [11].

FDA Adverse Event Reporting System (FAERS) is a pharmacovigilance system established by US FDA for the purpose of monitoring the safety of approved drug products. Adverse events of various drugs were continuously submitted by healthcare professionals and consumers to FAERS on a voluntary basis. Each case report provides various clinical information, such as the drugs used and the adverse reactions reported by the patient. Over these years, FAERS supported not only the drug safety surveillance of FDA, but also academic research about drug safety and development [12–15]. In view of the increasingly extensive application of FAERS data, the openFDA initiative (https://open.fda.gov/) was launched in June 2014 to provide convenient and computer-readable information of adverse events [16]. With openFDA, researchers were enabled to efficiently download and preprocess the FAERS data, so as to examine the association between drugs and adverse reactions.

In this analysis, we pooled adverse event reports submitted by non-small cell lung cancer patients to FAERS from 2004 to 2015. By comparing the patients exposed to carboplatin and other drugs, we examined whether hyponatremia and hypokalemia were significantly more concurrent with carboplatin therapy, so as to understand the effects of carboplatin on serum electrolyte levels. In addition, we explored the association between carboplatin and dehydration, which indicated the potential physiological mechanism of carboplatin-induced electrolyte abnormality.

## RESULTS

### Pooled analysis of hyponatremia adverse events

**Figure 1:**
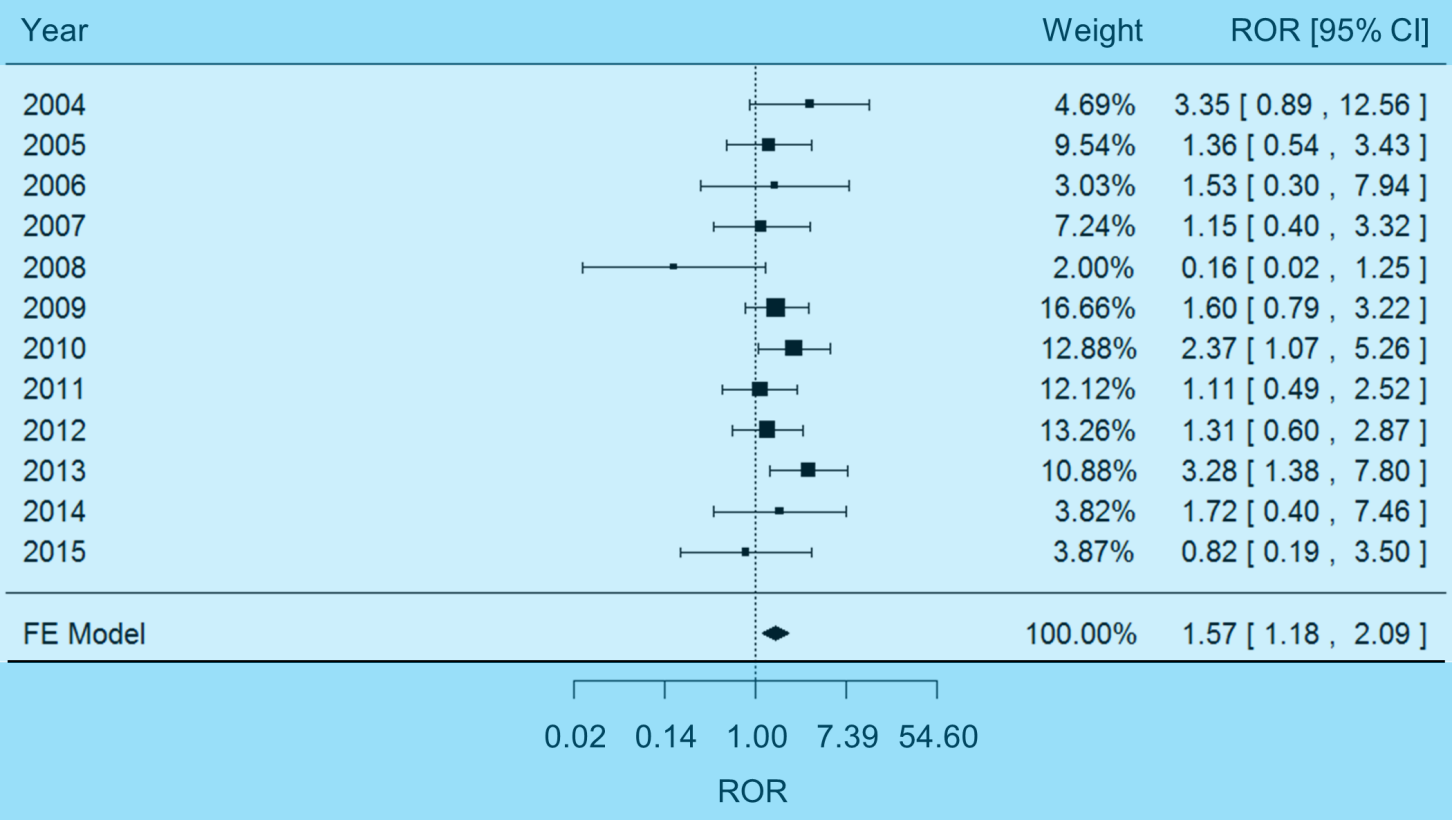
Forest plot of fixed-effects (FE) meta-analysis on hyponatremia adverse events

The pooled analysis on hyponatremia involved a total of 19901 adverse event reports (see Materials and Methods), among which 3907 reports (19.63%) were related to patients exposed to carboplatin. The χ^2^-based Q test [17] indicated no significant heterogeneity underlying the FAERS reports of different years (*P* = 0.37, I^2^ = 8%), so a fixed-effects model was selected to estimate the weighted average of effect size. The pooled reporting odds ratio (ROR) [18] proved to be significantly higher than 1.00 (Figure 1), suggesting that carboplatin therapy was associated with hyponatremia among non-small cell lung cancer patients (pooled ROR = 1.57, 95% CI 1.18-2.09, *P* = 1.99×10^-3^). There was no evidence of reporting bias in the above results, since the Egger's test [19] validated the symmetry of funnel plot (Supplementary Figure 1, *P* = 0.23).

### Pooled analysis of hypokalemia adverse events

**Figure 2:**
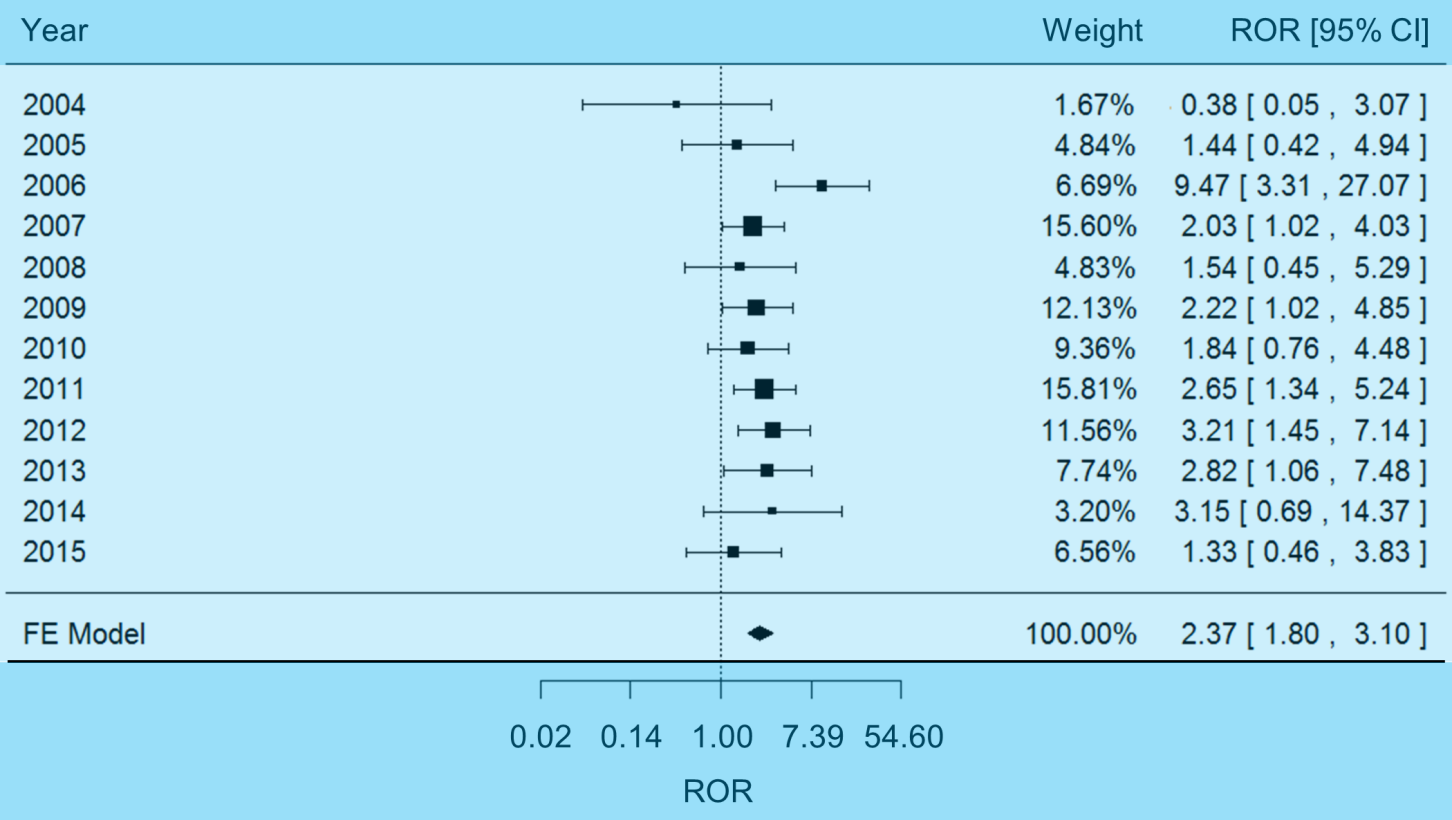
Forest plot of fixed-effects (FE) meta-analysis on hypokalemia adverse events

The FAERS reports were also analyzed for hypokalemia adverse events. No significant heterogeneity between different years was detected with the χ^2^-based Q test (*P* = 0.27, I^2^ = 18%), so a fixed-effects model was selected to pool the data. Combining effect sizes of different years as an outcome lead to a pooled ROR significantly higher than 1.00 (Figure 2), indicating the association between carboplatin therapy and hyponatremia (pooled ROR = 2.37, 95% CI 1.80-3.10, *P* = 5.24×10^-10^). No significant reporting bias was observed with Egger's test, namely, the shape of funnel plot did not show evident asymmetry (Supplementary Figure 2, *P* = 0.36).

### Physiological mechanism of carboplatin-induced electrolyte abnormality

**Figure 3:**
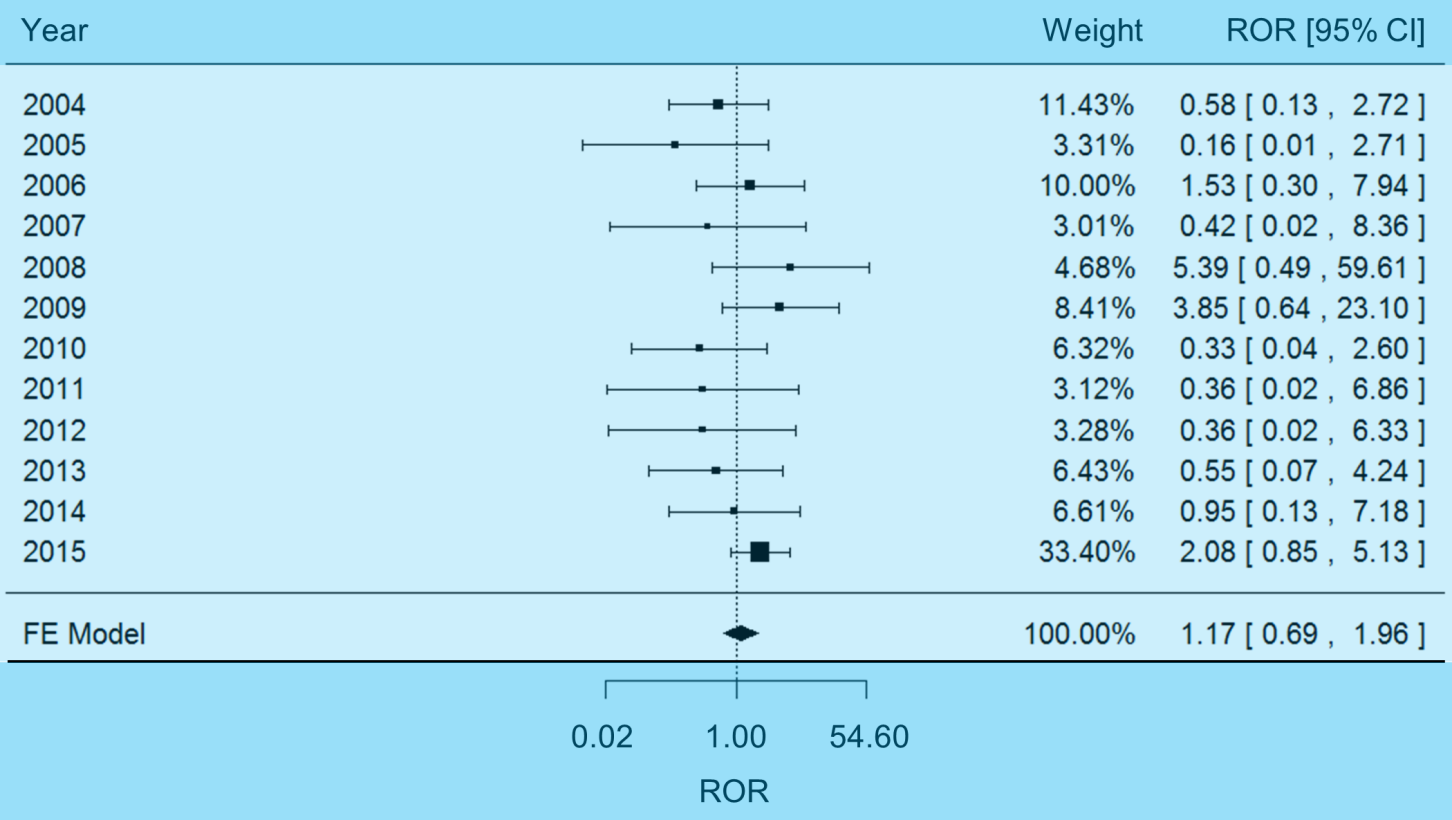
Forest plot of fixed-effects (FE) meta-analysis on impaired renal function

**Figure 4:**
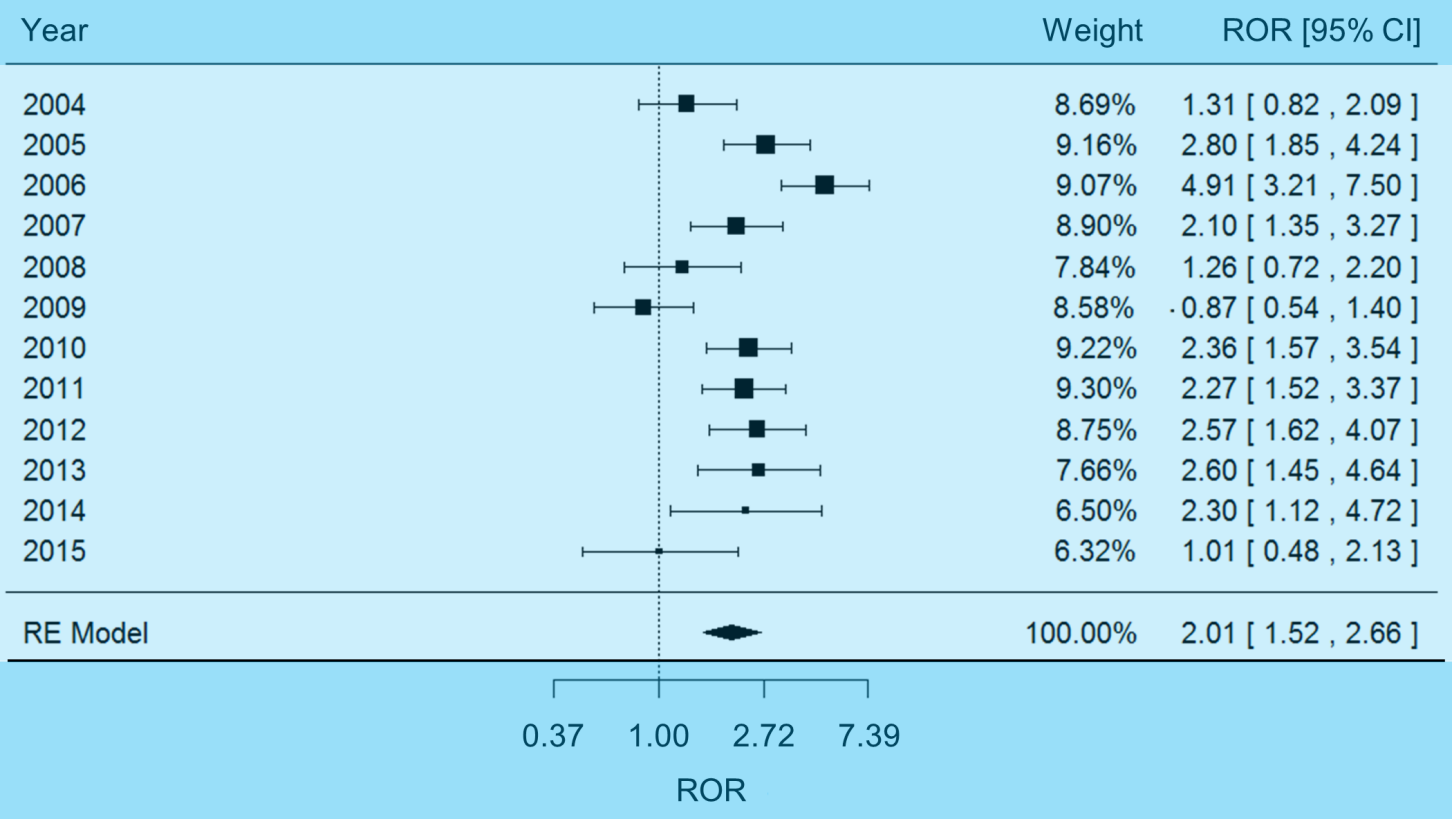
Forest plot of random-effects (RE) meta-analysis on dehydration adverse events

The above analysis on adverse events demonstrated the risk of carboplatin-induced electrolyte imbalance, but may not explain the underlying physiological mechanism. It is widely recognized that electrolyte abnormality, as a disorder of water homeostasis, may develop most often with impaired capability of the kidney to excrete free water [20] or excessive intake of water [21]. Therefore, we further examined the effects of carboplatin on renal function and water ingestion. On one hand, a fixed-effects meta-analysis (χ2-based Q test *P* = 0.41, I2 = 3%) indicated a non-significant association between carboplatin and impaired renal function (Figure 3, pooled ROR = 1.17, 95% CI 0.69-1.96). On the other hand, a random-effects meta-analysis (χ^2^-based Q test *P* = 1.09×10^-5^, I^2^ = 74%) demonstrated that carboplatin was significantly associated with dehydration (Figure 4, pooled ROR = 2.01, 95% CI 1.52-2.66, *P* = 8.37×10^-7^). And no reporting bias was found with regard to such an association (Supplementary Figure 3, Egger's test *P* = 0.22). These results suggested that carboplatin-induced dehydration may trigger strong sensation of thirst and naturally prompt overdrinking. In consequence, the electrolytes in body will be diluted by excess water.

## DISCUSSION

Although multiple disease states may contribute to the development of electrolyte disorders, various drugs have also been proved to interfere with the homeostasis of electrolytes [22–24]. As a class of severe and life-threatening reactions, drug-induced electrolyte abnormality is characterized by considerable morbidity and mortality [25], which may pose challenges to the clinicians who are not familiar with it. Strategies to address this issue involve careful consideration of risk factors during the process of treatment. In particular, early awareness of the adverse effects of certain drug products on serum electrolyte concentrations will facilitate a rational clinical management of cancer patients.

Carboplatin is becoming commonly used as an anti-cancer agent and increasingly noticed for potential safety risks. As an example, the association between carboplatin and electrolyte abnormality has been detected in clinical trials with limited samples and individual case reports. However, due to the lack of large-scale data of drug adverse events, the true incidence of electrolyte abnormality among general population exposed to carboplatin has yet to be clear. Therefore, in the present study, we retrieved 19901 reports of non-small cell lung cancer patients from FAERS. Compared to other therapies, carboplatin was significantly associated with hyponatremia and hypokalemia. In this case, the serum electrolyte concentrations of patients receiving carboplatin therapy should be continuously monitored for safety.

In addition to the above findings, the underlying physiological mechanism of carboplatin-induced electrolyte imbalance was also explored. We hypothesized that carboplatin may affect either normal renal function or water ingestion. First of all, we found that carboplatin was not likely to impair renal function. This result was consistent with the information in the FDA-approved drug label of carboplatin that “development of abnormal renal function test results is uncommon” among patients receiving carboplatin [26]. On the other hand, the FAERS reports pointed to the significant association between carboplatin and dehydration. It is common sense that water balance in human body is maintained via thirst [27]. Carboplatin-induced dehydration can enhance the thirst perception, which may lead to rapid and excessive drinking [28]. As a result, the serum electrolyte concentrations will be diluted by excess water [29, 30]. Therefore, in the course of carboplatin therapy, water ingestion should be controlled to avoid serious overhydration. Since this information has not been mentioned in the FDA-approved drug label of carboplatin, our results provided important references for clinicians to improve the clinical management of non-small cell lung cancer.

In spite of the rich information provided by FAERS reports, some limitations of current results need to be kept in mind and addressed in subsequent research. First, although FAERS reports indicate that carboplatin is associated with hyponatremia, hypokalemia and dehydration, such associations should be interpreted as cause-and-effect with caution. More clinical and toxicological research will be required to make a stronger argument for a cause-and-effect relationship and avoid false positive results. Second, since all FAERS reports are spontaneously submitted, the integrity of the data may not be fully warranted. As a result, detailed information about drug dosage is missing in many reports. It has been reported that a higher daily dose is proved to be a significant risk factor for drug toxicity [31]. Therefore, we expect further clinical studies to be conducted to investigate how the dosage factor may influence the risk of electrolyte abnormality. Third, besides non-small cell lung cancer, carboplatin is also widely used to treat other types of cancer, such as ovarian cancer [32] and bladder cancer [33]. Given the adverse effects encountered by non-small cell lung cancer patients, we suggest that more studies should be performed in different pathological conditions.

To our best knowledge, this is the first pharmacovigilance data analysis regarding carboplatin-induced electrolyte abnormality. In conclusion, both hyponatremia and hypokalemia were found to be significantly associated with carboplatin. In addition, excessive intake of water after carboplatin-induced dehydration may be an important factor affecting serum electrolyte levels. Such information will facilitate clinical management and prompt intervention of life-threatening electrolyte imbalance in the course of cancer treatment. Subsequent cohort studies and intensive toxicological experiments remain necessary to confirm and update our results.

## MATERIALS AND METHODS

### Raw data extraction

The original adverse events restored in FAERS were queried through openFDA platform according to the official tutorial (https://open.fda.gov/api/reference/). A total of 19901 adverse events of non-small cell lung cancer patients submitted between 2004 and 2015 were specified with the drug indication term “NON-SMALL CELL LUNG CANCER”. These data were retrieved by two independent researchers, reviewed by a third researcher, and finalized by the whole research team. Among these adverse events, those related to carboplatin were specified with the drug generic name “CARBOPLATIN”. And the adverse events of hyponatremia, hypokalemia, impaired renal function and dehydration were specified using the terms “HYPONATRAEMIA”, “HYPOKALAEMIA”, “RENAL IMPAIRMENT” and “DEHYDRATION”, respectively. All the above terms were coded using MedDRA terminology (http://www.meddra.org/).

### Statistical analysis

The adverse events of each year between 2004 and 2015 were used to construct a two-by-two contingency table as shown below, in which subjects were classified by carboplatin exposure (exposed or not exposed) and adverse reaction of interest (reported or not reported). The comparison between carboplatin and other drugs in the two-by-two table was summarized by the reporting odds ratio (ROR), which was calculated as (n_11_ × n_00_)/(n_10_ × n_01_). An ROR greater than 1.00 indicated a higher risk of carboplatin relative to other therapies for non-small cell lung cancer.

**Table:** 

Number of Adverse Events	Adverse Reaction Reported	Adverse Reaction Not Reported
Exposed to Carboplatin	*n_11_*	*n_10_*
Not Exposed to Carboplatin	*n_01_*	*n_00_*

Then, the RORs of different years were pooled together along with the corresponding 95% confidence intervals (CIs), so as to estimate the overall risk of carboplatin. Such a meta-analysis was conducted and visualized using ‘metafor’ package (https://cran.r-project.org/web/packages/metafor/) of R software. Heterogeneity of different years was examined with the χ^2^-based Q test, and *P*-value < 0.05 was considered significant. I^2^ statistics was calculated as the proportion of total heterogeneity contributed by between-year variation. A random-effects model (the DerSimonian and Laird method) was selected to pool the data if significant heterogeneity was observed. Otherwise, a fixed-effects model (the Mantel-Haenszel method) was selected.

In case of bias that may be introduced in the process of reporting adverse events and combining data of individual years, we used the funnel plot to assess the validity of meta-analysis. The asymmetry of funnel plot was examined by the method of Egger's linear regression test. *P* < 0.05 was considered to indicate statistically significant reporting bias.

## SUPPLEMENTARY MATERIALS FIGURES


