# Supplementary material for: Incidence and physiological mechanism of carboplatin-induced electrolyte abnormality among patients with non-small cell lung cancer
Source: Oncotarget. 2016 Oct 21;8(11):18417–23. doi: 10.18632/oncotarget.12813 (PMC5392339; doi:10.18632/oncotarget.12813)
Supplement: Supplementary file 1 [file oncotarget-08-18417-s001.pdf]

Incidence and physiological mechanism of carboplatin-induced electrolyte abnormality among patients with non-small cell lung cancer

Supplementary Material

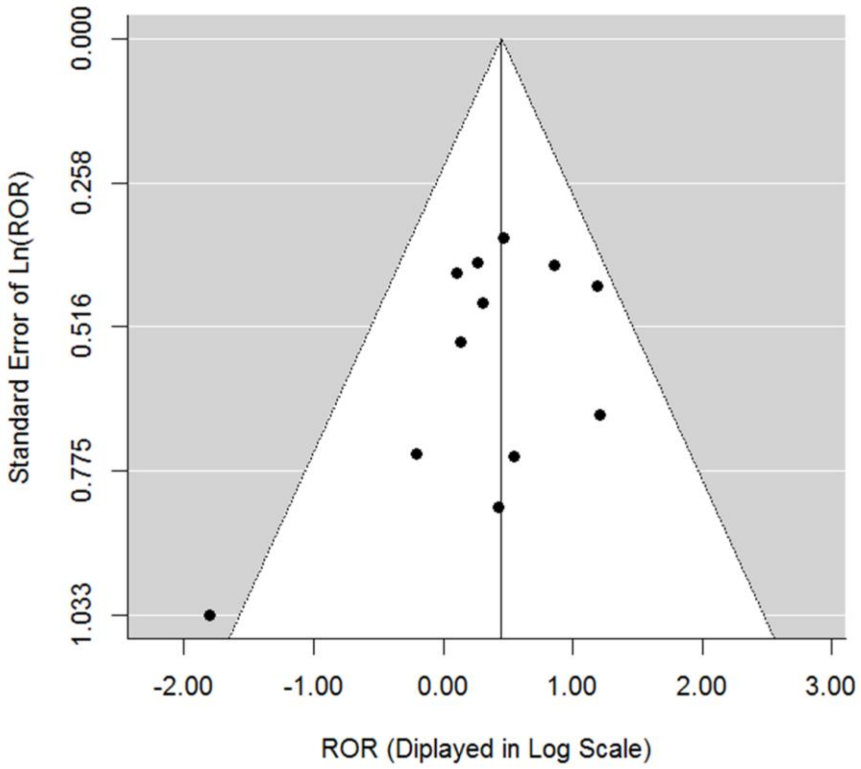

Supplementary Figure 1. Funnel plot for detecting reporting bias of hyponatremia adverse events.

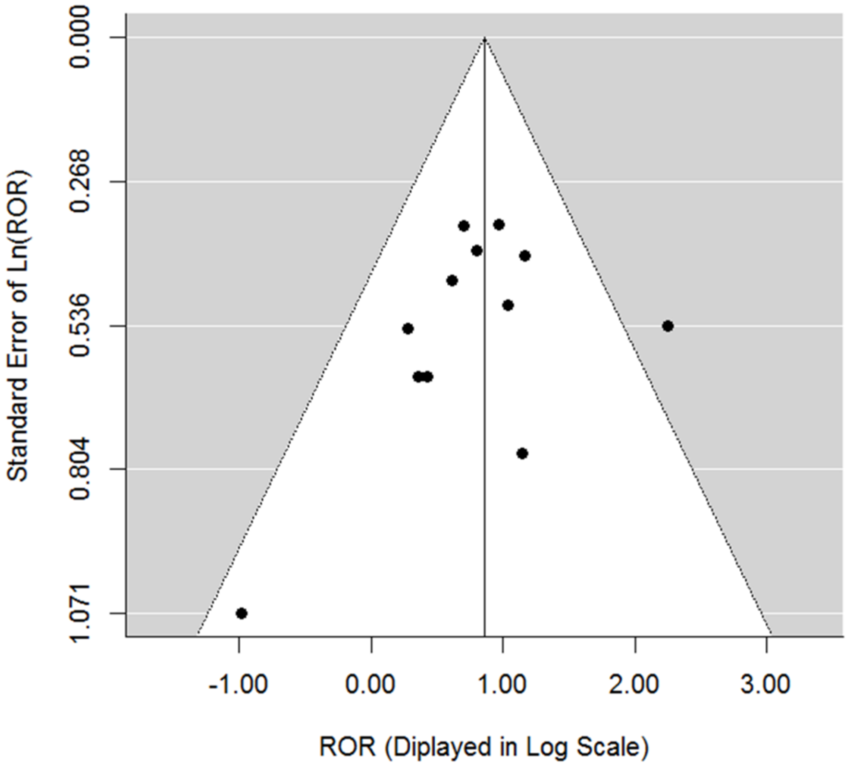

Supplementary Figure 2. Funnel plot for detecting reporting bias of hypokalemia adverse events.

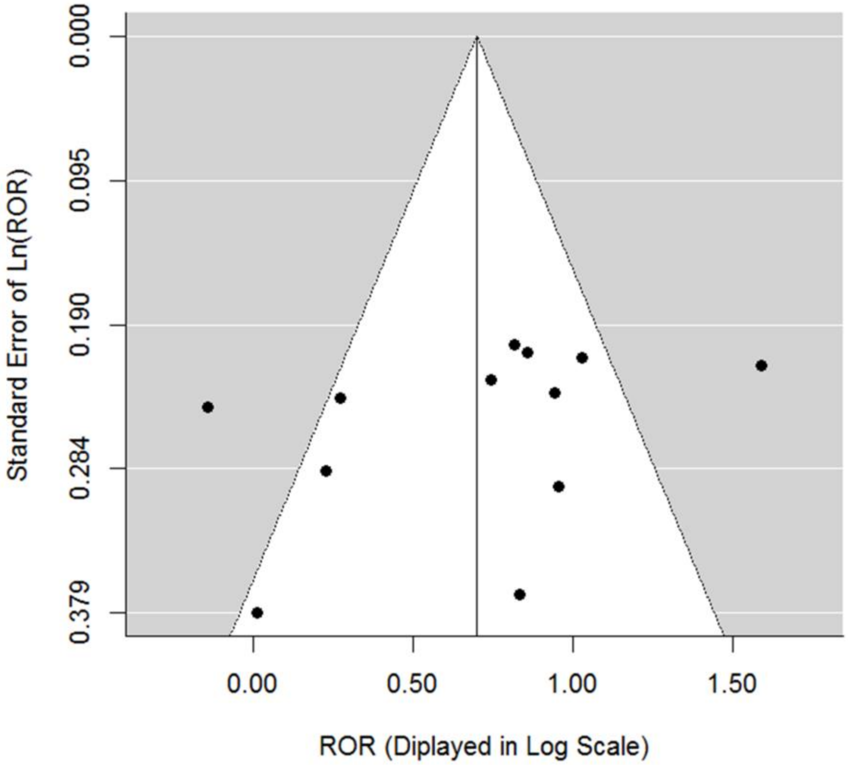

Supplementary Figure 3. Funnel plot for detecting reporting bias of dehydration adverse events.
